# Rupture in Polycystic Kidney Disease Presented as Generalized Peritonitis with Severe Sepsis: A Rare Case Report

**DOI:** 10.1155/2013/927676

**Published:** 2013-02-28

**Authors:** Muhammed Zahir, Hassan Al Muttairi, Surjya Prasad Upadhyay, Piyush N. Mallick

**Affiliations:** ^1^Al Jahra Hospital, Ministry of Health, Kuwait; ^2^Department of Anaesthesia and Critical Care, Al Jahra Hospital, Ministry of Health, Kuwait; ^3^Critical Care Unit, Al Jahra Hospital, Ministry of Health, Kuwait

## Abstract

Recurrent upper urinary tract (renal) infections have been reported to be frequent in patients with autosomal dominant polycystic kidney disease and often difficult to treat. Female preference and enteric organism predominance suggest that these renal infections are acquired to retrograde from the lower urinary tract. We encountered a rare case of bilateral polycystic kidneys with spontaneous intraperitoneal rupture of multiple infected renal cysts causing generalized peritonitis leading to severe sepsis with multiorgan failure. The patient is successfully managed with nephrectomy followed by prolonged supportive care in intensive care unit.

## 1. Introduction

Multiple renal cysts occur commonly in autosomal dominant Polycystic kidney disease and are generally considered to have little clinical significance [1, 2]. There are several complications that can develop with ADPKD including abdominal pain, renal masses, kidney stones, haematuria, hypertension, urinary tract infections, manifestations of stroke secondary to cerebral hemorrhage of ruptured aneurysms, hepatic cysts, cardiovascular system manifestations, and inguinal, abdominal, and umbilical hernias, as well as colonic diverticula [2–5]. Infection of a cyst within a polycystic kidney is a serious complication of ADPKD, potentially leading to abscess, sepsis, and death. Female preference and enteric organism predominance suggest that these renal infections are acquired as retrograde infection from the urinary bladder [6]. Rupture of an infected renal cyst is uncommon. Retroperitoneal sepsis and peritonitis due to rupture of infected cyst are very unusual. To our knowledge, early diagnosis and surgical treatment are crucial to prevent lethal complications.

## 2. Case Report

**Figure 1:**
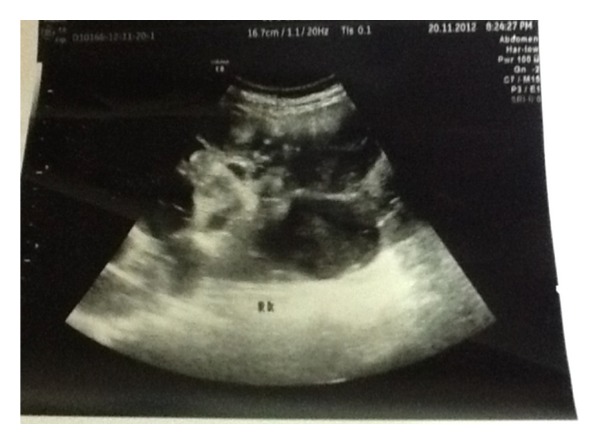
Ultrasonography image showing infected cyst.

A 52-year-old middle eastern female known to have bilateral polycystic kidneys with end stage renal failure on hemodialysis was admitted to emergency room with severe abdominal pain, distension, high-grade fever (38.7°C), features of early septicemia with tachycardia, and hypotension. Examination revealed generalized abdominal distension with severe tenderness, rigidity, and guarding suggestive of generalized peritonitis. White blood cell count was 24000/mm3, with 92% neutrophil, and arterial blood gas showed mild acidosis with pH 7.12 and lactate of 5.4 mmol/dL. Provisional diagnosis of peritonitis with septic shock was made, and the patient was resuscitated as per sepsis guideline. Plain X-ray abdomen showed only dilated bowel loops. Ultrasound abdomen revealed markedly enlarged bilateral polycystic kidneys with turbid fluid in most of the cysts (Figure 1).

**Figure 2:**
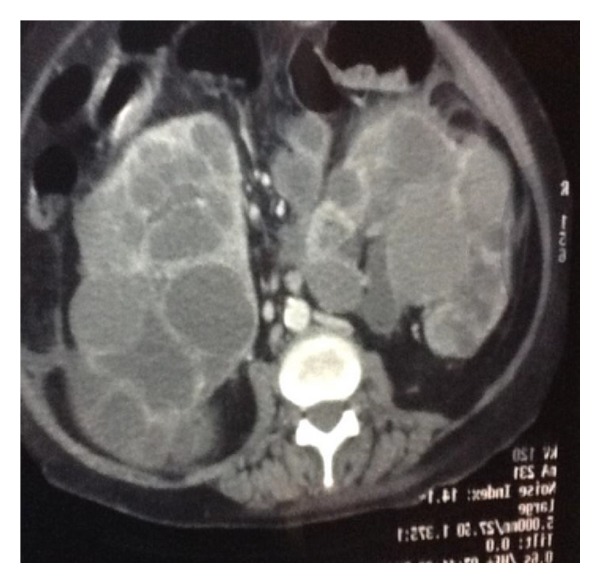
CT image showing bilateral renal cyst.

 CT abdomen showed hugely enlarged cystic kidneys extending up to the iliac fossae with fluid around it with minimal intraperitoneal fluid (Figure 2). 

**Figure 3:**
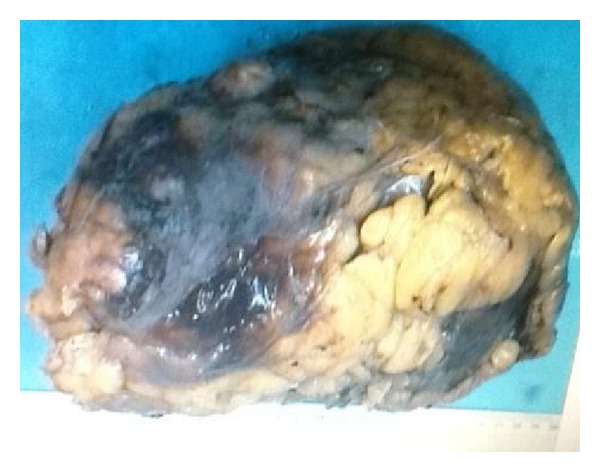
Postnephrectomy specimen (Right kidney).

**Figure 4:**
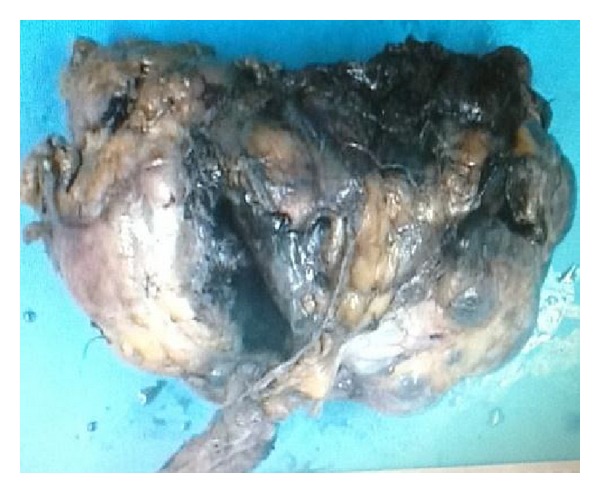
Postnephrectomy specimen showing rupture cyst.

Diagnostic laparotomy was performed after initial stabilization which revealed frank pus in the peritoneal cavity extending from the right posterolateral as well as from the lower pole of the right kidney with rupture of multiple infected renal cysts; hence, we proceeded to right simple nephrectomy. Postnephrectomy specimen with infected cyst is shown below (Figures 3 and 4).

The patient was shifted to Intensive Care Unit (ICU) for further management. Culture from the peritoneal pus as well as urine and blood culture were positive for *E. coli*. Postoperative course of the patient was complicated by severe sepsis with multiorgan failure (MOF) in the form of Adult Respiratory Distress Syndrome (ARDS), Disseminated Intravascular Coagulopathy (DIC). Patient was managed in ICU with ventilator, haemodialysis, and other supportive care, organ function gradually recovered, and the patient was shifted to ward on the 13th postoperative day. Further course in the ward was uneventful and patient was discharged home on the 20th postoperative day.

## 3. Discussion

Polycystic kidney disease (PKD or PCKD, also known as polycystic kidney syndrome) is a cystic genetic disorder of the kidneys [1]. There are two types of PKD: autosomal dominant polycystic kidney disease (ADPKD) and the less-common autosomal recessive polycystic kidney disease (ARPKD) which is characterized by the presence of multiple cysts (hence, “polycystic”) typically in both kidneys; however 17% of cases initially present as unilateral disease, with most cases progressing to bilateral disease in adulthood [2]. The cysts are numerous and are fluid-filled, resulting in massive enlargement of the kidneys. The disease can also damage the liver, pancreas, and, in some rare cases, the heart and brain. The two major forms of polycystic kidney disease are distinguished by their patterns of inheritance. Polycystic kidney disease is one of the most common life-threatening genetic diseases, affecting an estimated 12.5 million people worldwide [6]. 

Autosomal dominant polycystic kidney disease (ADPKD) is the most common one of all the hereditary cystic kidney diseases with an incidence of 1 to 2 : 1,000 live births [2, 4]. Studies show that 10% of end-stage renal disease (ESRD) patients being treated with hemodialysis were initially diagnosed and treated for ADPKD. ADPKD does not appear to demonstrate a preference for any particular ethnicity [2]. 

ADPKD is multisystem and progressive disease characterized by progressive multiple-cyst development in kidneys and other organs (liver, spleen, and pancreas). Cyst formation begins in utero from any point along the nephron although fewer than 5% of nephrons are thought to be involved. As the cysts accumulate fluid, they enlarge, separate entirely from the nephron, compress the neighboring renal parenchyma, and progressively compromise function [4, 7]. 

Most of the patients are asymptomatic in childhood, and symptoms are developed by 3rd decade with flank pain, gradual worsening of renal function, or complications of polycystic disease [4, 7]. 

Abdominal ultrasound and computed tomography (CT) scans can help in diagnosing PCKD and its associated complication such as infected cyst, peritoneal abscesses and increased amounts of peritoneal fluid. MRI and PET scans have been shown to be helpful in detecting infected cysts [6–8]. 

There are several complications which can develop with ADPKD including abdominal pain, renal masses, kidney stones, hematuria, hypertension, urinary tract infections, manifestations of stroke secondary to cerebral hemorrhage of ruptured aneurysms, hepatic cysts, cardiovascular system manifestations, and inguinal, abdominal, and umbilical hernias, as well as colonic diverticula [2–4, 8]. Infection of a cyst within a polycystic kidney is a serious complication of ADPKD, potentially leading to abscess, sepsis, and death. Female preference and enteric organism predominance suggest that these renal infections are acquired to retrograde from the urinary bladder [6]. Clinical presentation is that of peritonitis. To our knowledge, there is only one reported case of rupture polycystic kidney causing peritonitis in the literature so far [9], and this is the second case that we are reporting. Surgery remains the important therapeutic modality after preoperative medical support including broad-spectrum parenteral antibiotic coverage, correction of potential electrolyte and coagulation abnormalities, and aggressive intensive care with hemodynamic, pulmonary, and renal replacement support. Elective preventive nephrectomy of the left kidney was discussed with the patient and relative and they agreed to have nephrectomy but only at the time of renal transplantation as the patient was waiting for renal transplantation. 

## 4. Conclusion

The intraperitoneal rupture of infected renal cyst in polycystic kidney disease is very unusual and potentially serious complication. Multidisciplinary approach with prompt diagnosis and aggressive surgical intervention with intensive care support is crucial for survival after such complications.
